# Fecal Metabolomics and Potential Biomarkers for Systemic Lupus Erythematosus

**DOI:** 10.3389/fimmu.2019.00976

**Published:** 2019-05-03

**Authors:** Qiong Zhang, Xiaofeng Yin, Haifang Wang, Xing Wu, Xin Li, Yao Li, Xiaohe Zhang, Chen Fu, Haixia Li, Yurong Qiu

**Affiliations:** ^1^Laboratory Medicine Center, Nanfang Hospital, Southern Medical University, Guangzhou, China; ^2^Longsee Biomedical Corporation, Guangzhou, China; ^3^Huayin Medical Laboratory Center Co., Ltd., Guangzhou, China

**Keywords:** feces, metabolomics, biomarker, systemic lupus erythematosus, liquid chromatography, mass spectrometry

## Abstract

The role of metabolomics in autoimmune diseases has been a rapidly expanding area in researches over the last decade, while its pathophysiologic impact on systemic lupus erythematosus (SLE) remains poorly elucidated. In this study, we analyzed the metabolic profiling of fecal samples from SLE patients and healthy controls based on ultra-high-performance liquid chromatography equipped with mass spectrometry for exploring the potential biomarkers of SLE. The results showed that 23 differential metabolites and 5 perturbed pathways were identified between the two groups, including aminoacyl-tRNA biosynthesis, thiamine metabolism, nitrogen metabolism, tryptophan metabolism, and cyanoamino acid metabolism. In addition, logistic regression and ROC analysis were used to establish a diagnostic model for distinguishing SLE patients from healthy controls. The combined model of fecal PG 27:2 and proline achieved an area under the ROC curve of 0.846, and had a good diagnostic efficacy. In the present study, we analyzed the correlations between fecal metabolic perturbations and SLE pathogenesis. In summary, we firstly illustrate the comprehensive metabolic profiles of feces in SLE patients, suggesting that the fecal metabolites could be used as the potential non-invasive biomarkers for SLE.

## Introduction

Systemic lupus erythematosus (SLE) is an autoimmune disease with persistent inflammation that affects multiple organ systems, characterized by high morbidity and low quality of life. The etiological factors and pathogenesis of SLE are still not completely understood (1). However, a lot of evidences suggest that the gut microbiome takes an important role in inflammatory and autoimmune diseases by affecting the immune system and metabolic pathways (2, 3).

Changes in microbiota are of great interest in SLE research as they may reveal novel insights into the etiopathogenesis or develop diagnostic markers for SLE disease. The researches on the gut microbiota compositions have exhibited lower *Firmicutes*, higher *Bacteroidetes* and lower *Firmicutes/Bacteroidetes* (F/B) ratio in SLE patients compared to healthy controls (4, 5). Additionally, further study have reported that the F/B ratio was strongly associated with serum free fatty acids (FFA) levels and the fecal short chain fatty acids levels (SCFA) paralleled those of serum FFA in healthy controls, whereas these associations were not found in lupus patients (6). SCFAs produced from bacterial fermentation of fiber have anti-inflammatory and immunomodulatory effects through the impact of regulatory T (Treg) cells (7). The metabolite profile provides a functional readout of microbial activity and can be used as an intermediate phenotype mediating host–microbiome interactions.

Although metabolomics are becoming a novel research hotspot in human diseases, little is known about the metabolic activity of patients with SLE and whether eventual differences might be related to the pathogenesis of the disease. Recently, a metabolomics study on SLE sera has shown evidence of profoundly dampened glycolysis, Krebs cycle, fatty acid oxidation and amino acid metabolism (8). Bengtsson et al. (9) have found an increased oxidative activity in SLE, supported by increased xanthine oxidase activity and an increased turnover in the urea cycle. However, there is no published study on the potential fecal metabolic dysbiosis associated with SLE.

In the current study, we investigated the metabolomics on fecal samples of SLE patients and healthy controls to analyze the correlations between fecal metabolic perturbations and SLE pathogenesis and to explore specific metabolites that could potentially be used as biomarkers of SLE patients.

## Methods

### Subjects and Sample Collection

The cohort consisted of 32 SLE patients, 26 healthy controls (HC) with matched age and gender. All patients fulfilled the American College of Rheumatology (ACR) classification criteria for SLE (9) and were recruited from the Department of Rheumatology, Nanfang Hospital, Guangdong, China. All the SLE patients had no history of other autoimmune diseases. And healthy controls who had no history of autoimmune disease were from Department of Health Management of Nanfang Hospital. All the participants were female and none of them were given an extreme diet. In order to avoid the extra influence of gut microbes on intestinal metabolites, all participants had no history of probiotic diets and antibiotics during the past 4 weeks. The protocol of this study was approved by the ethics committee of Nanfang Hospital. Written informed consents were obtained from all subjects before enrollment in the study. Fresh fecal samples (around 400mg) were collected within 1 week after enrollment, suspended in stool storage solution according to the manufacturer's protocol of Longseegen stool storage kit (Longsee Biomedical Corporation, Guangzhou, China) and stored at −80°C.

### Sample Preparation

Feces were homogenized and 200 μL of fecal sample was dried under vacuum. Then 800 μL of methanol was added into the fecal samples. All of the samples were shaken for 30 s and subjected to ultrasound for 10 min. Then the mixture was incubated at −20°C for 2 h to facilitate protein precipitation. The mixtures were then centrifuged at 13,000 rpm at 4°C for 15 min and the supernatants were collected, followed by vacuum drying and re-dissolved with 200 μL of methanol /water (1:1, v/v). The supernatants were subjected to metabolomics profiling by ultrahigh performance liquid chromatography and mass spectrometry (UHPLC-MS).

### Instrumentation and Analytical Conditions

Sample analysis was performed by Ekspert UltraLC 110 (AB Sciex, Framingham, MA, USA) coupled with Triple TOF 5600+ (AB Sciex, Framingham, MA, USA). ACQUITY UPLC HSS T3 column (2.1 mm ×100 mm, 1.8 μm, Waters, USA) was used for chromatographic separation. The column temperature was 35 °C. The mobile phase A consisted of a 1:9 acetonitrile/water (v/v) solution containing 0.1% formic acid and the mobile phase B consisted of a 9:1 acetonitrile/water (v/v) solution containing 0.1% formic acid. The flow rate was kept at 0.3 mL/min during a 33-min run with the following gradient: 100% A from 0 to 4 min, 100% A from 4 to 6 min, 75% A from 6 to 25min, 100% B from 25 to 29 min, 100% B from 29 to 31 min and 100% A from 31 to 33 min. All samples were analyzed in positive mode. Parameters of mass spectrometry were as follows: ion spray voltage of 5,500 V, curtain gas of 40 Pa, source temperature of 550°C, collision energies for collision-induced dissociation of 30 eV, the MS1 scan range of 50–1,000 *m/z* and the MS2 scan range of 25–1,000 *m/z*.

### Metabolites Identification

All putative identities were confirmed by matching with entries in the MassBank of North America (MoNA) (http://mona.fiehnlab.ucdavis.edu/), the HMDB database (http://www.hmdb.ca) and KEGG database (http://www.genome.jp/kegg) using the molecular mass data (m/z) of samples. The metabolite would be identified on the condition that a mass difference between observed value and the database value was <0.025Da.

### Metabolomics Data Analysis

The datasets were analyzed by pattern recognition methods using MetaboAnalyst 3.0. To reduce the concentration differences between samples, the data were normalized to the total spectral peak height and log transformations were used for non-linear conversions of the data to make the skewed distributions more symmetric. Auto scaling aims to make each variable comparable to each other. For univariate analysis, the statistical significance of features was determined between SLE group and HC group, using *t*-test by MetaboAnalyst 3.0. *P* < 0.05 was considered to be statistically significant. For multivariate statistical analysis, partial-least-squares discrimination analysis (PLS-DA) was applied to eliminate the effect of inter-subject variability among the participants and identify metabolites that significantly contributed to the classification, and was validated based on the multiple correlation coefficient (R^2^), and cross-validated R^2^ (Q^2^) by 10-fold cross validation to ensure the quality of the multivariate model and to avoid the risk of overfitting. Metabolites were ranked according to their variable importance in the projection (VIP) scores from PLS-DA model and usually metabolites with VIP scores >1.0 are considered as the significant contributors.

To identify the most relevant metabolic pathways involved in SLE, a metabolomics pathway analysis was employed by MetaboAnalyst 3.0. We assessed both a test for overrepresentation of altered metabolites within a pathway (hypergeometric tests) (10) and for the impact of the changed metabolites on the function of the pathway through alterations in critical junction points of the pathway (relative betweenness centrality) (11). The results of each of the 80 human pathways of KEGG were simultaneously plotted to show the most significant pathways in terms of hypergeometric test *p*-values and impact.

Receiver-operating characteristic curve (ROC) analysis was carried out to evaluate the diagnostic performance of individual metabolites (12) by MetaboAnalyst 3.0 and MedCalc software version 13.0.6.0 (Broekstraat, Mariakerke, Belgium). A stepwise binary logistic-regression was used to establish a combined model. To address the issue of potential over-fitting in generating ROC curves from the combined model, 100-fold cross validation and permutation test were used to calculate the performance by MetaboAnalyst 3.0 via ROC curve based model evaluation (Tester) section (13, 14).

## Results

### Basic Characteristics of Participants

The demographics of SLE patients at the time-point of sample collection are presented in Table 1, where also data on levels of complements, C reactive protein (CRP), autoantibodies, as well as medication treatments. There were no significant differences in age (*p* = 0.648) and BMI (*p* = 0.367) between the SLE group and HC group by two-tailed unpaired Student's *t*-test.

**Table 1:** Characteristics of the study population.

	**SLE**	**HC**
Fecal samples	32	26
Female	100%	100%
Age, years, mean ± SD	39.44 ± 15.45	41.15 ± 12.33
BMI, kg/m^2^, mean ± SD	23.08 ± 3.90	22.15 ± 3.82
**DISEASE ACTIVITY PARAMETERS**		
ESR, mm/h, mean (median)	24.94 (17.50)	
CRP, mg/L, mean (median)	3.21 (0.95)	
SLEDAI, median (range)	6 (0–16)	
**AUTOANTIBODY STATUS**		
Positive anti-dsDNA	31.25%	
Positive anti- Sm	21.88%	
Positive ANA	93.75%	
**TREATMENT**		
Glucocorticoid	28	
Hydroxycholorquine	21	
Cyclophosphamide	8	
Leflunomide	4	

*BMI, body mass index; ESR, erythrocyte sedimentation rate; CRP, C reactive protein; SLEDAI, systemic lupus erythematosus disease activity index*.

### Overall Metabolomics Analysis of Fecal Samples

**Figure 1:**
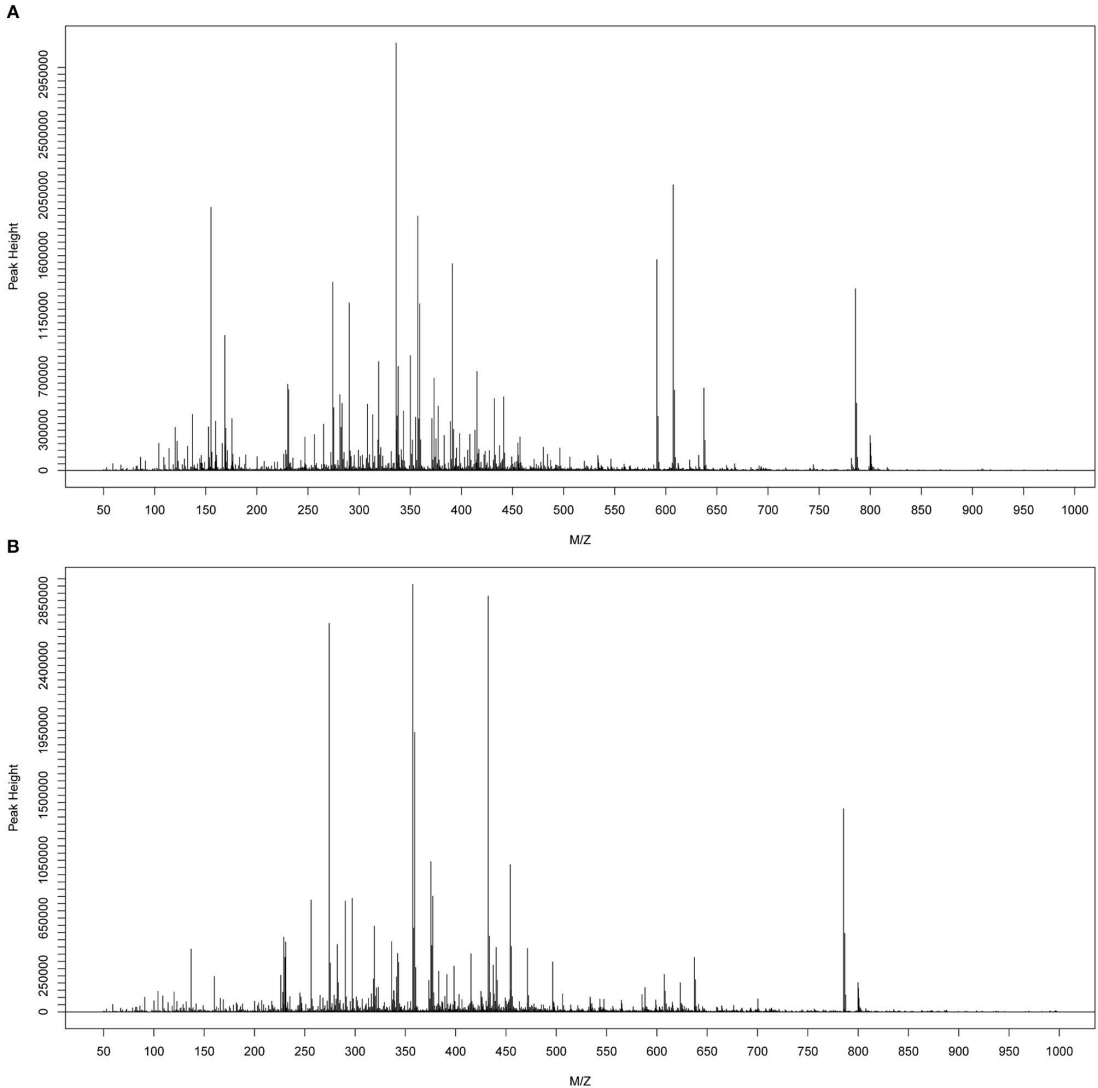
Typical mass spectra of the SLE group **(A)** and HC group **(B)**.

**Figure 2:**
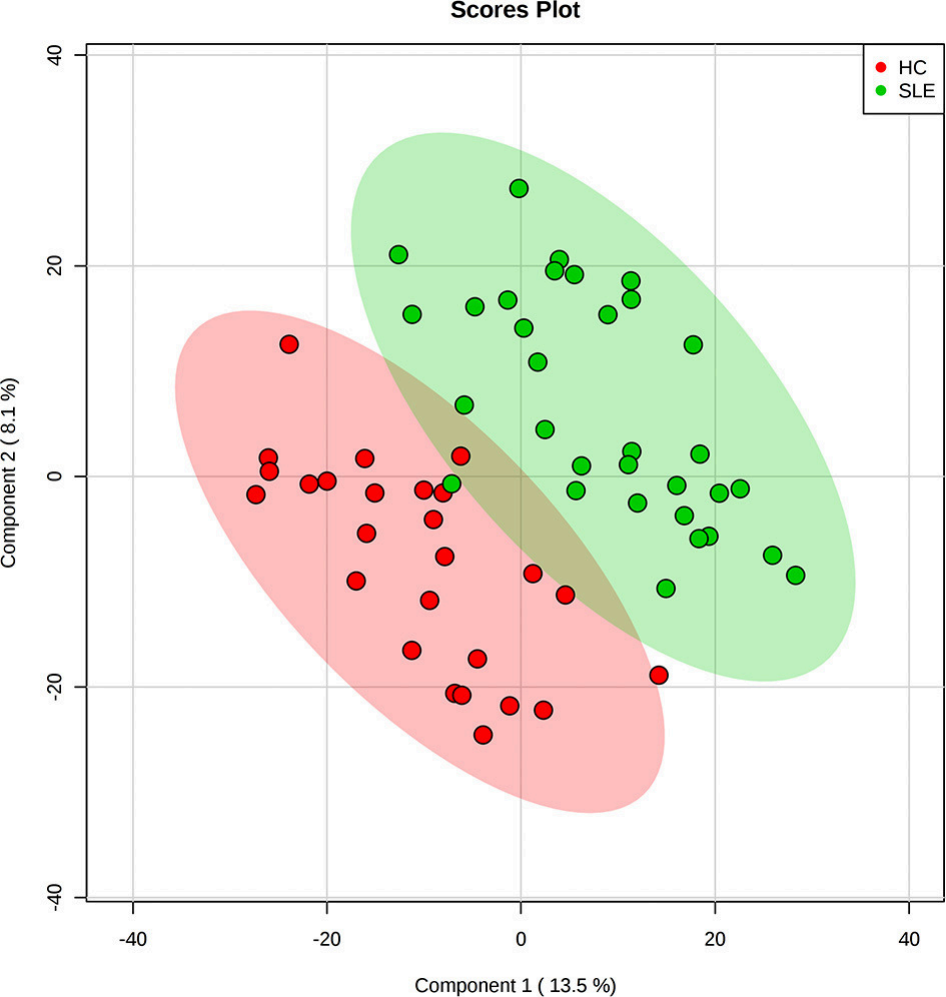
Partial least squares discriminant analysis (PLS-DA) of fecal metabolomics data from SLE patients and healthy controls. Fecal metabolites distinguished SLE patients from healthy controls. The green dots represented SLE patients and the red dots represented healthy controls in the two-dimensional PLS-DA score plots.

**Figure 3:**
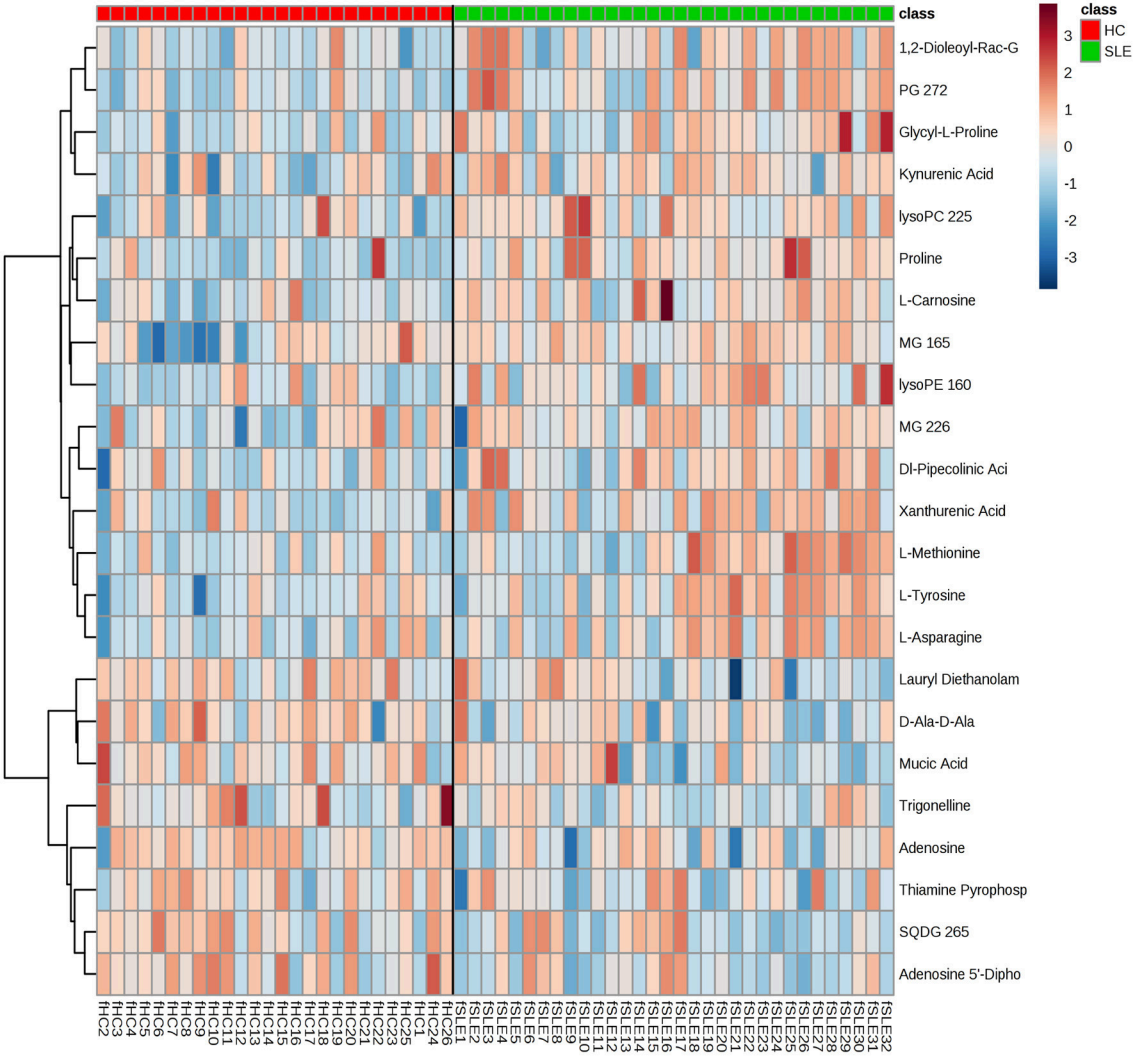
Metabolic patterns in SLE patients and healthy controls. Fecal metabolite profiles in SLE patients and healthy controls were shown as heatmaps. Each row represented data for a specific metabolite and each column represented an individual. Different colors corresponded to the different intensity level of metabolites. Red and blue colors represented increased and decreased levels of metabolites, respectively.

**Figure 4:**
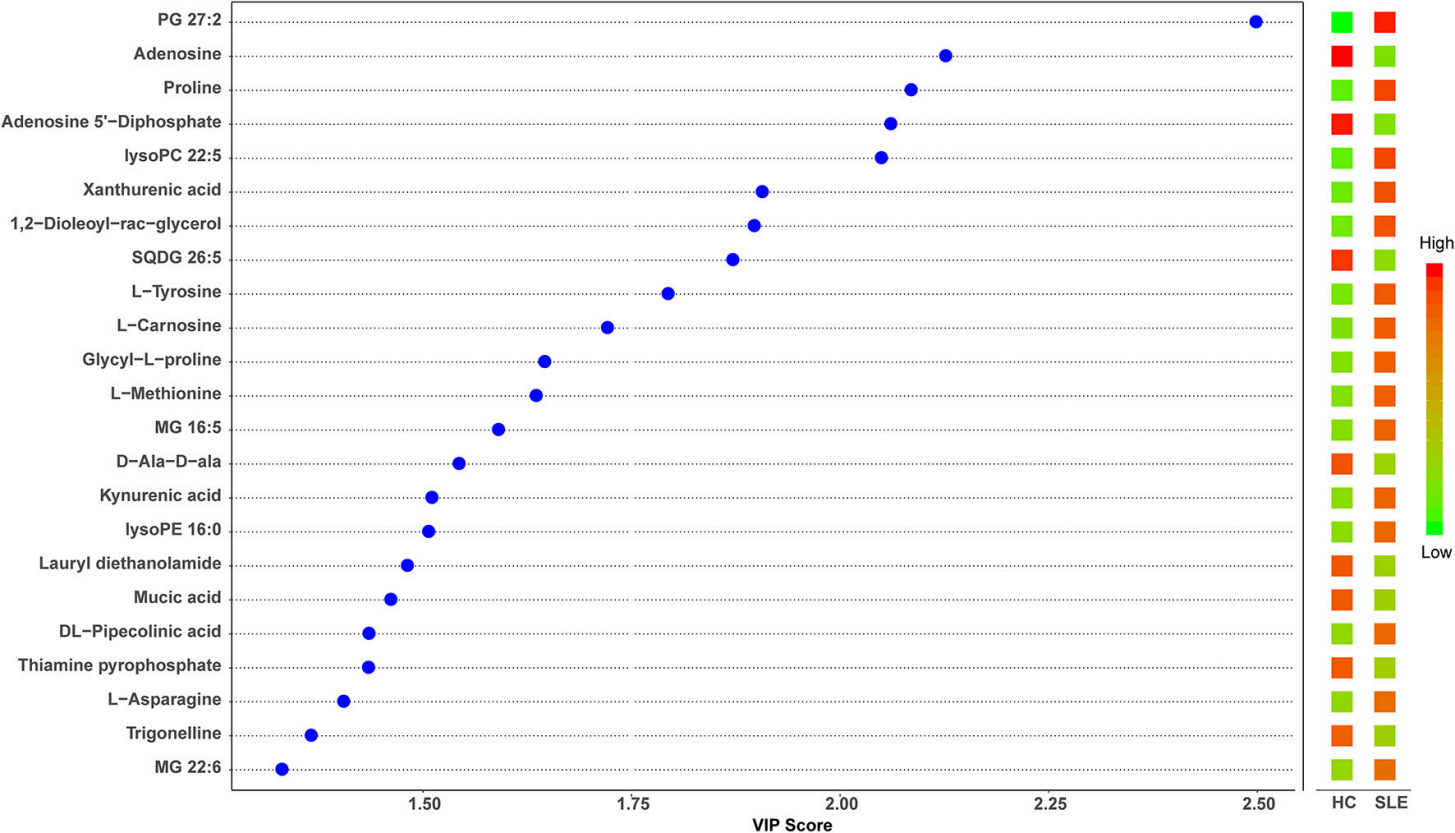
Partial least squares discriminant analysis (PLS-DA) variable importance in projection (VIP) plot of significantly differential metabolites in SLE patients and healthy controls. The χ-axis represented the VIP scores, and the *y-*axis represented the compounds. Red and green colors represented increased and decreased levels of metabolites, respectively.

The metabolomes of 58 fecal samples from SLE patients and healthy controls were characterized and compared. Representative mass spectrum of SLE patients and healthy controls were shown in Figures 1A,B. A total of 3,924 molecular features were obtained and subjected to statistical analysis using MetaboAnalyst 3.0. PLS-DA revealed that HC and SLE samples exhibited differential distributions with a Q^2^ of 0.53 and a R^2^ of 0.98, which indicated that the model was not overlifting and was reliable (Figure 2). Features with VIP scores > 1.0 in multivariate statistical analysis and *p* < 0.05 in univariate analysis were considered as the most significant metabolites and were visualized through heatmap (Figure 3). A total of 23 most significant metabolites that were involved in the amino acids, lipids, purine, and vitamin metabolisms were altered in feces of SLE group, compared with HC group (Table 2). The levels of proline, L-tyrosine, L-methionine, L-asparagine, Dl-pipecolinic acid, Glycyl-L-proline, xanthurenic acid, kynurenic acid, L-carnosine, monoacylglycerol (MG) 22:6, MG 16:5, lysophosphatidylethanolamine (lysoPE) 16:0, lysophosphatidylcholine (lysoPC) 22:5, phosphatidylglycerol (PG) 27:2 and 1,2-dioleoyl-rac-glycerol were increased in fecal samples of SLE. Whereas, the levels of adenosine, adenosine 5'-diphosphate (ADP), D-Alaninyl-D-alanine (D-Ala-D-Ala), lauryl diethanolamide, sulfoquinovosyl diacylglyceride (SQDG) 26:5, thiamine pyrophosphate, trigonelline, and mucic acid were decreased in SLE (Figure 4).

**Table 2:** Fecal identified differential metabolites between SLE patients and healthy controls.

**Metabolite**	***p***	**VIP**	**FC**	**Pathways**
Proline	0.002	2.08	1.94	Amino acid metabolism
L-Tyrosine	0.007	1.79	3.50	Amino acid metabolism
L-Methionine	0.015	1.63	3.27	Amino acid metabolism
L-Asparagine	0.037	1.40	2.78	Amino acid metabolism
Dl-Pipecolinic acid	0.033	1.43	1.50	Amino acid metabolism
Glycyl-L-Proline	0.014	1.64	2.30	Amino acid metabolism
D-Ala-D-ala	0.022	1.54	0.63	Amino acid metabolism
L-Carnosine	0.010	1.72	2.03	Amino acid metabolism
Xanthurenic acid	0.004	1.90	2.20	Amino acid metabolism
Kynurenic acid	0.025	1.51	1.39	Amino acid metabolism
Lauryl diethanolamide	0.028	1.48	0.75	Fatty acid metabolism
1,2-Dioleoyl-Rac-Glycerol	0.004	1.90	3.64	Glycerolipid metabolism
MG 22:6	0.049	1.33	1.14	Glycerolipid metabolism
MG 16:5	0.018	1.59	1.14	Glycerolipid metabolism
SQDG 26:5	0.005	1.87	0.56	Glycerolipid metabolism
lysoPE 16:0	0.025	1.51	1.87	Glycerophospholipid metabolism
lysoPC 22:5	0.002	2.05	2.00	Glycerophospholipid metabolism
PG 27:2	0.000	2.50	4.33	Glycerophospholipid metabolism
Adenosine	0.001	2.12	0.54	Purine metabolism
Adenosine 5'-Diphosphate	0.002	2.06	0.35	Purine metabolism
Trigonelline	0.043	1.36	0.35	Vitamin metabolism
Thiamine pyrophosphate	0.033	1.43	0.74	Vitamin metabolism
Mucic acid	0.030	1.46	0.74	Other

*VIP, variable importance in the projection; FC, fold change; D-Ala-D-ala, D-Alanyl-D-alanine; MG, monoacylglycerol; SQDG, sulfoquinovosyl diacylglyceride; lysoPE, lysophosphatidylethanolamine; lysoPC, lysophosphatidylcholine; PG, phosphatidylglycerol*.

### Metabolic Pathway Analysis

**Figure 5:**
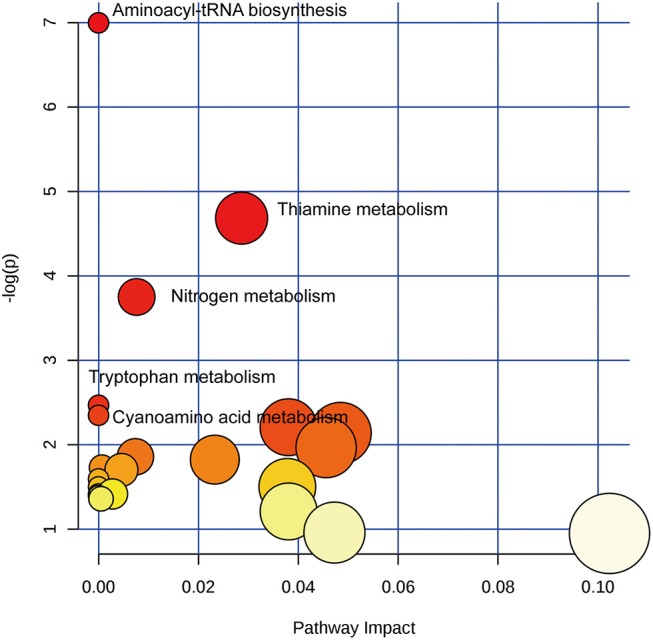
Pathway analysis of altered metabolites isolated from SLE patients compared with healthy controls. Twenty Three metabolic pathways were enriched in fecal samples. Aminoacyl-tRNA biosynthesis, thiamine metabolism, nitrogen metabolism, tryptophan metabolism, and cyanoamino acid metabolism significantly disturbed compared with healthy controls (*p* < 0.1). The χ-axis represented the pathway impact, and the *y-*axis represented the –log (*p*).

To identify biologically meaningful patterns based on the metabolomics data in feces, pathway analysis was performed through the KEGG metabolic library using Metaboanalyst 3.0 (15, 16). The perturbed metabolic pathways in the fecal samples were shown in Supplementary Table S1, depicting aminoacyl-tRNA biosynthesis, thiamine metabolism, nitrogen metabolism, tryptophan metabolism, and cyanoamino acid metabolism as significantly enriched in SLE patients compared with healthy controls (*p* < 0.1, Figure 5). The increased L-asparagine, L-methionine, L-tyrosine, and L-proline were hit in aminoacyl-tRNA biosynthesis. Meanwhile, L-tyrosine, L-asparagine were also enriched in Nitrogen metabolism. The increased L-tyrosine and decreased thiamine pyrophosphate were involved in thiamine metabolism. Additionally, the increased kynurenic acid and xanthurenic acid participated in tryptophan metabolism.

### Diagnostic Performance of Metabolites

**Figure 6:**
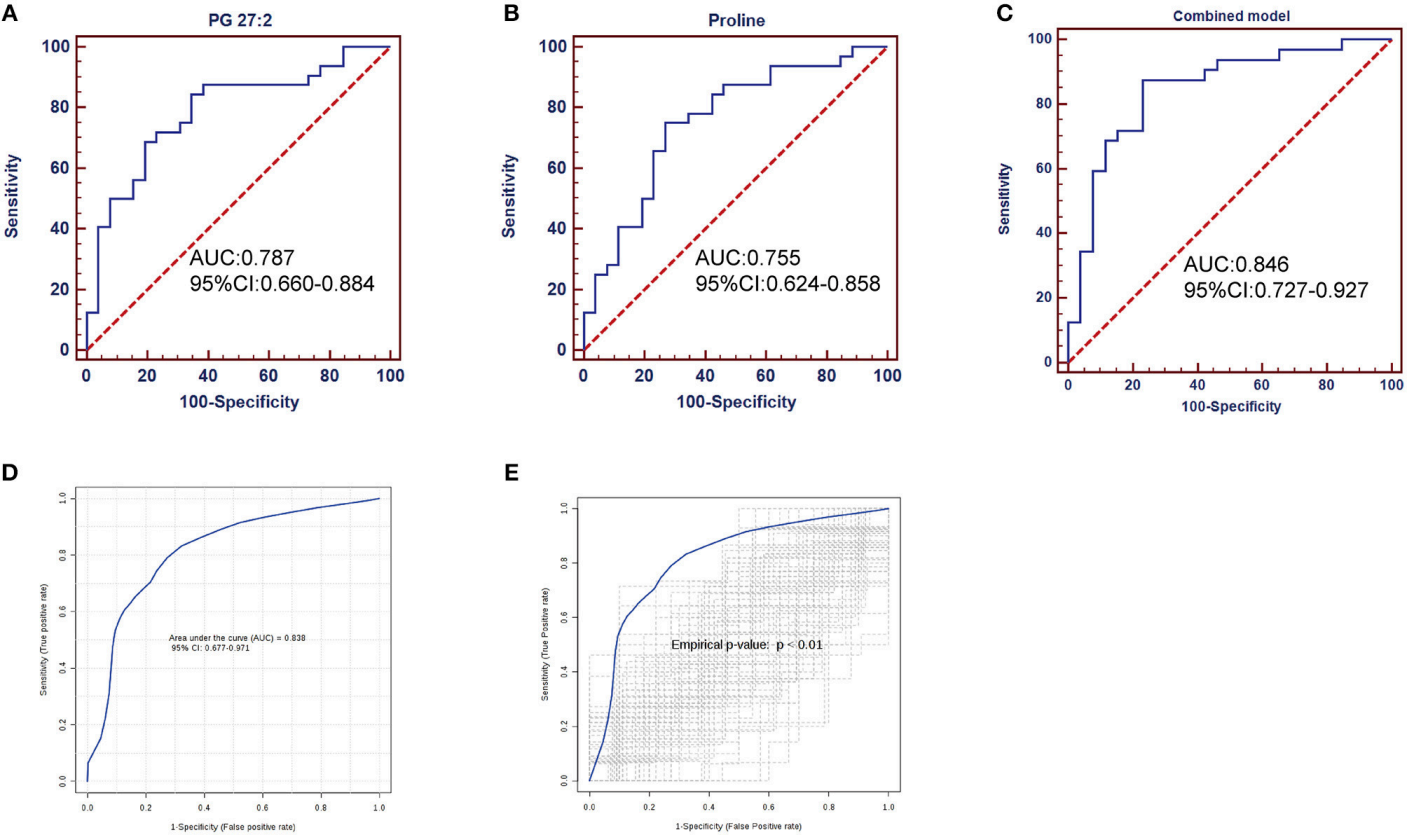
ROC analysis of potential biomarkers for differentiating SLE patients from healthy controls. PG 27:2 showed an AUC of 0.787 (95% CI: 0.660–0.884, *p* = 0.0002) **(A)**; the proline presented an AUC of 0.755 (95% CI: 0.624–0.858, *p* = 0.0009) **(B)**; the combined model performed an AUC of 0.846 (95% CI: 0.727–0.927, *p* < 0.0001) **(C)**; the combined model was evaluated by 100-fold cross validation **(D)** and permutation test **(E)**, achieving an AUC of 0.838 (95% CI: 0.677–0.971, *p* < 0.0001) and a *p* < 0.01.

Having established that the metabolomics is instrumental in differentiating SLE patients and healthy controls, we wanted to explore potential diagnostic biomarkers for SLE. The top 15 significant metabolites ranked with VIP scores were chosen for classical univariate ROC curve analysis by MetaboAnalyst 3.0 to evaluate their diagnostic efficacy preliminarily. The diagnostic performances of these metabolites were shown in Supplementary Table S2. The area under the curve (AUC) values of PG 27:2, adenosine, proline, adenosine 5'-diphosphate, lysoPC 22:5, xanthurenic acid, 1, 2-dioleoyl-rac-glycerol, SQDG 26:5, and L-carnosine were 0.787, 0.748, 0.755, 0.732, 0.754, 0.716, 0.730, 0.716 and 0.715, respectively. To improve the diagnostic performance of SLE, a combined model was established through the top 15 metabolites, equated as Logit (P) = 0.423+1.186 ^*^PG 27:2+1.136^*^ proline for fecal samples. The corresponding ROC curve of PG 27:2, proline and the combined model were shown in Figure 6. PG 27:2 had an AUC of 0.787 (95% confidence interval (CI):0.660–0.884) with the sensitivity of 84.4% and the specificity of 65.4% at the cutoff of −0.5911 (Figure 6A). The proline presented an AUC of 0.755 (95% CI: 0.624–0.858) with the sensitivity of 75.0% and the specificity of 73.1% at the cutoff of −0.2980 (Figure 6B). And the combined model achieved an AUC of 0.846 (95% CI: 0.727–0.927) with the sensitivity of 87.5% and the specificity of 76.9% (Figure 6C). Through a 100-fold cross validation, the combined model remained an AUC value of 0.838 (95% CI: 0.677–0.971) (Figure 6D) and had a *p* < 0.01 following 100 permutation tests (Figure 6E). These results demonstrated that there was no over-fitting issue and the combined model was reliable. Besides, the increased fecal levels of PG 27:2 and proline were correlated with increased risk of SLE (odds ratio (OR): 3.28, 95% CI: 1.54–6.96; OR: 3.12, 95% CI: 1.26–7.69, respectively).

## Discussion

To the best of our knowledge, this is the first published report on fecal metabolomics in SLE. The metabolite profiles of fecal samples allowed differentiation of SLE patients from healthy controls. Twenty three fecal metabolites were significantly altered in SLE patients. Aminoacyl-tRNA biosynthesis, thiamine metabolism, nitrogen metabolism, tryptophan metabolism, and cyanoamino acid metabolism were perturbed in SLE compared to healthy controls. Furthermore, the combined diagnosis of PG 27:2 and proline was of greatest importance to distinguish SLE patients from healthy controls.

In the present study, several amino acids were changed in SLE patients. The levels of fecal xanthurenic acid and kynurenic acid were elevated in SLE patients, which are catabolites of tryptophan through the kynurenine pathway. Besides, the tryptophan metabolism was significantly enriched. The enhanced tryptophan degradation and accumulation of kynurenine in sera and plasm have been observed in SLE patients (17, 18). Perl et al. (19) performed a quantitative metabolome study of peripheral blood lymphocytes and indicated accumulation of kynurenine and kynurenic acid in SLE patients, which could be reversed by N-acetylcysteine (NAC) treatment. They also found kynurenine stimulated mechanistic target of rapamycin (mTOR) activity in CD4^−^CD8^−^ double-negative T cells in healthy control. As previous study shown, NAC improved lupus disease activity by blocking mTOR in T cells (20). Therefore, kynurenine could contribute to mTOR activation and may be the metabolic target of the therapeutic action by NAC in patients with SLE. Additionally, kynurenine has the potential to act as a predictor of NAC effect in SLE. Meanwhile, the kynurenine pathway is an effective mechanism in modulating the immune response and in inducing immune tolerance (21). These findings implicated that kynurenine pathway or tryptophan metabolism may play an important role in SLE development and is a potential therapeutic target for SLE. It is noteworthy that glucogenic amino acids, like proline, L-methionine and L-asparagine, and glucogenic and ketogenic amino acid, L-tyrosine, were increased in feces of SLE patients, indicating that there might be disorders in glucose metabolism and energy metabolism as these amino acids could emerge as potential energy sources. Whereas concentrations of several amino acids, including asparagine, tyrosine and proline, were decreased in the serum samples of SLE patients, (8, 22, 23) though in the publication by Yan et al. methionine was up-regulated (23). Proline in feces is major components of most colonic epithelium mucus glycoproteins (24), and the increased proline in fecal samples may reflect an alteration in the production and function of mucins covering the gut epithelium. A study on intestinal microbes of rheumatoid arthritis revealed that the abundance of *Collinsella* correlated with high levels of asparagine. *Collinsella* increased gut permeability and enhanced disease severity in experimental arthritis mice (25). Hence, these findings may suggest the association of amino acids and the intestinal function. We speculate the amino acid absorption may be insufficient, resulting in certain amino acids accumulation in gut and lower levels of amino acids in serum. The most significantly enriched aminoacyl-tRNA biosynthesis which was hit by L-asparagine, L-methionine, L-tyrosine and proline reflected the amino acid turnover or protein biosynthesis in SLE patients.

As previous study shown, the vitamin B6 were significantly reduced in sera samples of SLE patients (8). We found the vitamin B metabolism was skewed in fecal samples of SLE patients. Trigonelline is a product of the metabolism of niacin (vitamin B3). Trigonelline has been proved to be effective in inhibiting intestinal microbial metabolism of choline, depicting blocking the formation of trimethylamine (TMA) from choline and further its conversion into proatherosclerotic metabolite, trimethylamine-N-oxide (TMAO) (26). Hence, trigonelline may be potential to alleviate the cardiovascular diseases of SLE patients. Moreover, trigonelline exerted robust antibacterial effect against many bacteria, such as *Escherichia coli, Proteus mirabilis* and *Enterococcus faecalis* (27).Thiamine pyrophosphate (TPP) is the active form of thiamine (vitamin B1). Costliow et al. demonstrated that in environments with limited thiamine availability, the biosynthesis of thiamine is essential for growth and competition of *B. thetaiotaomicron* (28), which is a species of bacterium of the genus *Bacteroides* and belongs to phylum Bacteroidetes in human gut flora. The higher *Bacteroidetes* and lower *Firmicutes/Bacteroidetes* (F/B) ratio have been reported in SLE patients (4, 5). We speculate that the decreased fecal trigonelline and thiamine pyrophosphate could play important roles in the persistence of microbes through vitamin B metabolism in the gut of SLE patients. In addition, it is notable that a link between the imbalance of T cell population and specific vitamins, such as vitamin A and vitamin D, has previously been described in patients with SLE (29–32). However, it is unclear whether vitamin B may have the similar immunomodulatory function. The regulation of vitamin B on immunity is also worth exploring in the pathogenesis of SLE, which may be helpful to explore new therapeutic target for SLE.

Metabolites involved in purine metabolism were down-regulated in patients with SLE compared to healthy controls, including ADP and adenosine. As a hydrolytic enzyme of adenosine, serum adenosine deaminase (ADA) activity in SLE patients was significantly increased and positively correlated with SLE disease activity (33), accounting for the reduction of adenosine to some extent. Adenosine is one of the major immunosuppressive factors generated by Treg cells for reducing responses to self, regulating tolerance to tissue grafts and preventing autoimmune diseases (34–36). The reduction number and defective function of Treg cells in SLE patients have also been reported in previous studies (37–39). Therefore, the decreased adenosine may be related to the imbalance of Treg cells and may act as a predictor of Treg population in SLE. Furthermore, adenosine can mediate intestinal epithelial restitution and anti-inflammation (40, 41). In line with our recent study on disordered intestinal microbes in SLE, by PICRUSt analysis, the purine metabolism was positively associated with the genus *Streptococcus* that was highly enriched in SLE patients (42), indicating the fecal purine metabolism as a functional readout of the gut microbiome in SLE patients. These findings supported that adenosine or purine metabolism could be involved in the pathogenesis of SLE by regulating systemic immunity, inflammation and gut microbes.

Several imbalances were also noted in the patients' lipid metabolism, including MG 22:6, MG 16:5, SQDG 26:5, PG 27:2, lysoPE 16:0, lysoPC 22:5, and 1,2-Dioleoyl-Rac-Glycerol. Zhou et al. (43) monitored 222 lipids belonging to 15 lipid species in serum in SLE and found that lipids were significantly changed. The level of lysoPCs with saturated carbon chains (from C16:0 to C20:0) presented higher level in healthy controls, whereas lysoPCs with unsaturated carbon chains (from C18:2 to C22:6) showed higher levels in the inactive SLE patients. Similarly, we found that except SQDG 26:5, the polyunsaturated lipids MG 22:6, MG 16:5, lysoPC 22:5, and PG 27:2 were elevated in SLE patients in our study. These observations implicated the potential linkage between carbon chains of lipids and SLE disease pathology. At the pathways level, these altered lipids in our study were involved in glycerolipid metabolism, glycerophospholipid metabolism and fatty acid metabolism, which was consistent with published studies (8, 43).

A large number of metabolomics studies have found potential biomarkers for several autoimmune diseases using plasma, serum, urine and synovial fluid samples (44–47). In fact, fecal sample is highly interesting since its collection is very simple and clearly non-invasive for the patients. A diagnostic model of SLE was established with the fecal PG 27:2 and proline in our study. The AUC of the optimized model was 0.846 (95% CI: 0.727–0.927). The combined model remains an AUC value of 0.838 (95%CI: 0.677–0.971) after 100-fold cross validation and had a *p* < 0.01 after 100 permutation tests. The diagnostic performance of the combined biomarker was reliable and was better than single feature. However, to ensure clinical utility of a biomarker, it might be necessary to include additional control groups, including patients with other autoimmune or inflammatory diseases with a larger samples size in the future study.

Of important note, metabolism can be varied by drug intake (48). Wu et al. (8) indicated some metabolites associated with the prednisone (glucocorticoid), such as 2-methylbutyroylcarnitine, citrulline, leucine, phenylalanine, urate, and valine. In our study, most of SLE patients were currently on treatment of glucocorticoid, hydroxycholorquine, cyclophosphamide and leflunomide, which ethically for patients' benefit could not be terminated. Therefore, the drug-intake factor could not be adjusted as in certain metabolic studies (8, 9, 22, 23). In the present study, principal component analysis (PCA) indicated that there were no significant differences in metabolic profiles at the overall level between patients who had received specific medicine treatment and those who had not (Supplementary Figure S1). However, the levels of lauryl diethanolamide, trigonelline, MG 22:6 and mucic acid were associated with glucocorticoid, hydroxycholorquine, cyclophosphamide and leflunomide treatment, respectively, by Mann-Whitney *U*-test (Supplementary Table S3). Thus, metabolites may be altered not only for disease itself, but also for treatment. A drawback of this study is a small sample size of SLE patients with no treatments to perfectly evaluate the medication effects on fecal metabolites. We are planning to further analyze treatment-naive SLE patients with a larger sample size to further identify specific metabolites among different treatments.

Taken together, we observed metabolic dysregulation with certain metabolic pathways in fecal samples of SLE patients and a novel metabolic model was proposed for the better diagnosis of SLE in a non-invasive way. It is challenging and necessary to estimate the causal relationship between metabolites and pathogenesis. Future prospective investigations with an expanding samples quantity are needed to validate the clinical value and accuracy of the present potential metabolic biomarkers in SLE diagnosis.

## Ethics Statement

Ethics approval for this project was obtained from the Ethics Committee of the Nanfang Hospital, Southern Medical University.

## Author Contributions

YQ, HL, and QZ conceived and designed the study, QZ, XY, HW, and XW performed the experiments and analyzed the data. XL and YL contributed to the sample collection and storage. XZ and CF were responsible for clinical data collection. QZ and XY wrote the manuscript. All authors approved the final version of the manuscript to be submitted.

### Conflict of Interest Statement

The authors declare that the research was conducted in the absence of any commercial or financial relationships that could be construed as a potential conflict of interest.
