# Supplementary material for: Fecal Metabolomics and Potential Biomarkers for Systemic Lupus Erythematosus
Source: Front Immunol. 2019 May 3;10:976. doi: 10.3389/fimmu.2019.00976 (PMC6509220; doi:10.3389/fimmu.2019.00976)
Supplement: Supplementary file 1 [file Data_Sheet_1.doc]

Supplementary Material

# Supplementary Tables

**Supplementary Table S1**. Pathway analysis in fecal samples of SLE and HC groups.

| Pathway analysis | Total | Expected | Hits | Raw p | -log(p) | Holm adjust | FDR | Impact |
| --- | --- | --- | --- | --- | --- | --- | --- | --- |
| Aminoacyl-tRNA biosynthesis | 75 | 0.46739 | 4 | 9.15E-04 | 7.00E+00 | 7.32E-02 | 7.32E-02 | 0.00E+00 |
| Thiamine metabolism | 24 | 0.14956 | 2 | 9.25E-03 | 4.68E+00 | 7.30E-01 | 3.70E-01 | 2.87E-02 |
| Nitrogen metabolism | 39 | 0.24304 | 2 | 2.35E-02 | 3.75E+00 | 1.00E+00 | 6.27E-01 | 7.63E-03 |
| Tryptophan metabolism | 79 | 0.49231 | 2 | 8.48E-02 | 2.47E+00 | 1.00E+00 | 1.00E+00 | 0.00E+00 |
| Cyanoamino acid metabolism | 16 | 0.099709 | 1 | 9.55E-02 | 2.35E+00 | 1.00E+00 | 1.00E+00 | 0.00E+00 |
| Purine metabolism | 92 | 0.57333 | 2 | 1.10E-01 | 2.21E+00 | 1.00E+00 | 1.00E+00 | 3.80E-02 |
| Citrate cycle (TCA cycle) | 20 | 0.12464 | 1 | 1.18E-01 | 2.14E+00 | 1.00E+00 | 1.00E+00 | 4.84E-02 |
| Alanine, aspartate and glutamate metabolism | 24 | 0.14956 | 1 | 1.40E-01 | 1.97E+00 | 1.00E+00 | 1.00E+00 | 4.56E-02 |
| Phenylalanine, tyrosine and tryptophan biosynthesis | 27 | 0.16826 | 1 | 1.56E-01 | 1.86E+00 | 1.00E+00 | 1.00E+00 | 7.38E-03 |
| beta-Alanine metabolism | 28 | 0.17449 | 1 | 1.61E-01 | 1.82E+00 | 1.00E+00 | 1.00E+00 | 2.33E-02 |
| Glycolysis or Gluconeogenesis | 31 | 0.19319 | 1 | 1.77E-01 | 1.73E+00 | 1.00E+00 | 1.00E+00 | 6.80E-04 |
| Pyruvate metabolism | 32 | 0.19942 | 1 | 1.82E-01 | 1.70E+00 | 1.00E+00 | 1.00E+00 | 4.59E-03 |
| Ubiquinone and other terpenoid-quinone biosynthesis | 36 | 0.22435 | 1 | 2.03E-01 | 1.60E+00 | 1.00E+00 | 1.00E+00 | 0.00E+00 |
| Butanoate metabolism | 40 | 0.24927 | 1 | 2.23E-01 | 1.50E+00 | 1.00E+00 | 1.00E+00 | 0.00E+00 |
| Valine, leucine and isoleucine degradation | 40 | 0.24927 | 1 | 2.23E-01 | 1.50E+00 | 1.00E+00 | 1.00E+00 | 3.78E-02 |
| Nicotinate and nicotinamide metabolism | 44 | 0.2742 | 1 | 2.42E-01 | 1.42E+00 | 1.00E+00 | 1.00E+00 | 0.00E+00 |
| Histidine metabolism | 44 | 0.2742 | 1 | 2.42E-01 | 1.42E+00 | 1.00E+00 | 1.00E+00 | 2.80E-03 |
| Ascorbate and aldarate metabolism | 45 | 0.28043 | 1 | 2.47E-01 | 1.40E+00 | 1.00E+00 | 1.00E+00 | 0.00E+00 |
| Phenylalanine metabolism | 45 | 0.28043 | 1 | 2.47E-01 | 1.40E+00 | 1.00E+00 | 1.00E+00 | 0.00E+00 |
| Lysine degradation | 47 | 0.2929 | 1 | 2.57E-01 | 1.36E+00 | 1.00E+00 | 1.00E+00 | 5.00E-04 |
| Cysteine and methionine metabolism | 56 | 0.34898 | 1 | 2.98E-01 | 1.21E+00 | 1.00E+00 | 1.00E+00 | 3.81E-02 |
| Tyrosine metabolism | 76 | 0.47362 | 1 | 3.83E-01 | 9.60E-01 | 1.00E+00 | 1.00E+00 | 4.72E-02 |
| Arginine and proline metabolism | 77 | 0.47985 | 1 | 3.87E-01 | 9.50E-01 | 1.00E+00 | 1.00E+00 | 1.02E-01 |

**Supplementary Table S2. The diagnostic performance of the top 15 metabolites with high VIP values.**

| Metabolite | VIP | AUC | 95% CI | Sensitivity | Specificity | Cutoffs |
| --- | --- | --- | --- | --- | --- | --- |
| PG 27:2 | 2.4967 | 0.787 | 0.660-0.884 | 84.4% | 65.4% | >-0.5911 |
| Adenosine | 2.1247 | 0.748 | 0.616-0.852 | 71.9% | 73.1% | <=0.2213 |
| Proline | 2.0833 | 0.755 | 0.624-0.858 | 75.0% | 73.1% | >-0.2980 |
| Adenosine 5'-Diphosphate | 2.0589 | 0.732 | 0.599-0.840 | 59.4% | 80.8% | <=-0.3546 |
| lysoPC 22:5 | 2.0476 | 0.754 | 0.623-0.857 | 81.2% | 69.2% | >-0.4224 |
| Xanthurenic acid | 1.9048 | 0.716 | 0.583-0.827 | 68.7% | 73.1% | >-0.2942 |
| 1,2-Dioleoyl-rac-glycerol | 1.8952 | 0.730 | 0.597-0.838 | 75.0% | 76.9% | >=-0.1701 |
| SQDG 26:5 | 1.8696 | 0.716 | 0.583-0.827 | 62.5% | 80.8% | <=-0.4350 |
| L-Tyrosine | 1.792 | 0.671 | 0.535-0.788 | 46.9% | 92.3% | >0.5142 |
| L-Carnosine | 1.7193 | 0.715 | 0.581-0.826 | 59.4% | 84.6% | >0.0828 |
| Glycyl-L-proline | 1.6441 | 0.681 | 0.546-0.798 | 78.1% | 61.5% | >-0.4925 |
| L-Methionine | 1.6339 | 0.653 | 0.516-0.773 | 53.1% | 80.8% | >0.2321 |
| MG 16:5 | 1.5887 | 0.632 | 0.495-0.755 | 96.9% | 34.6% | >-0.5943 |
| D-Ala-D-Ala | 1.5415 | 0.695 | 0.560-0.809 | 81.2% | 57.7% | <=0.3242 |
| Kynurenic acid | 1.5089 | 0.686 | 0.551-0.802 | 68.7% | 73.1% | >0.2496 |

VIP, variable importance in the projection; AUC, area under the ROC curve

**Supplementary Table S3. Relationship of fecal metabolites and clinical treatment.**

|  | Glucocorticoid | Hydroxycholorquine | Cyclophosphamide | Leflunomide |
| --- | --- | --- | --- | --- |
| Proline | p=0.721 | p=0.457 | p=0.404 | p=0.209 |
| L-Tyrosine | p=0.209 | p=0.081 | p=0.104 | p=0.151 |
| L-Methionine | p=0.891 | p=0.367 | p=0.334 | p=0.361 |
| L-Asparagine | p=0.169 | p=0.155 | p=0.104 | p=0.457 |
| Dl-Pipecolinic acid | p=0.527 | p=0.208 | p=0.848 | p=0.564 |
| Glycyl-L-Proline | p=0.640 | p=0.133 | p=0.623 | p=0.934 |
| D-Ala-D-ala | p=0.120 | p=0.907 | p=0.334 | p=0.564 |
| L-Carnosine | p=0.680 | p=0.411 | p=0.564 | p=0.392 |
| Xanthurenic acid | p=0.457 | p=0.133 | p=0.881 | p=0.805 |
| Kynurenic acid | p=0.763 | p=0.725 | p=0.915 | p=0.424 |
| Lauryl diethanolamide | **p=0.047** | p=0.223 | p=0.273 | p=0.361 |
| 1,2-Dioleoyl-Rac-Glycerol | p=0.361 | p=0.168 | p=0.313 | p=0.332 |
| MG 22:6 | p=0.934 | p=0.577 | **p=0.041** | p=0.602 |
| MG 16:5 | p=0.680 | p=0.907 | p=0.848 | p=0.188 |
| SQDG 26:5 | p=0.230 | p=0.755 | p=0.685 | p=0.305 |
| lysoPE 16:0 | p=0.106 | p=0.725 | p=0.815 | p=0.491 |
| lysoPC 22:5 | p=0.057 | p=0.208 | p=0.254 | p=0.188 |
| PG 27:2 | p=0.680 | p=0.434 | p=0.815 | p=0.457 |
| Adenosine | p=0.680 | p=0.506 | p=0.949 | p=0.721 |
| Adenosine 5'-Diphosphate | p=0.072 | p=0.876 | p=0.535 | p=0.209 |
| Trigonelline | p=0.564 | **p=0.027** | p=0.623 | p=0.978 |
| Thiamine pyrophosphate | p=0.763 | p=0.845 | p=0.949 | p=0.072 |
| Mucic acid | p=0.721 | p=0.254 | p=0.273 | **p=0.038** |

# Supplementary Figures

**Supplementary Figure S1**


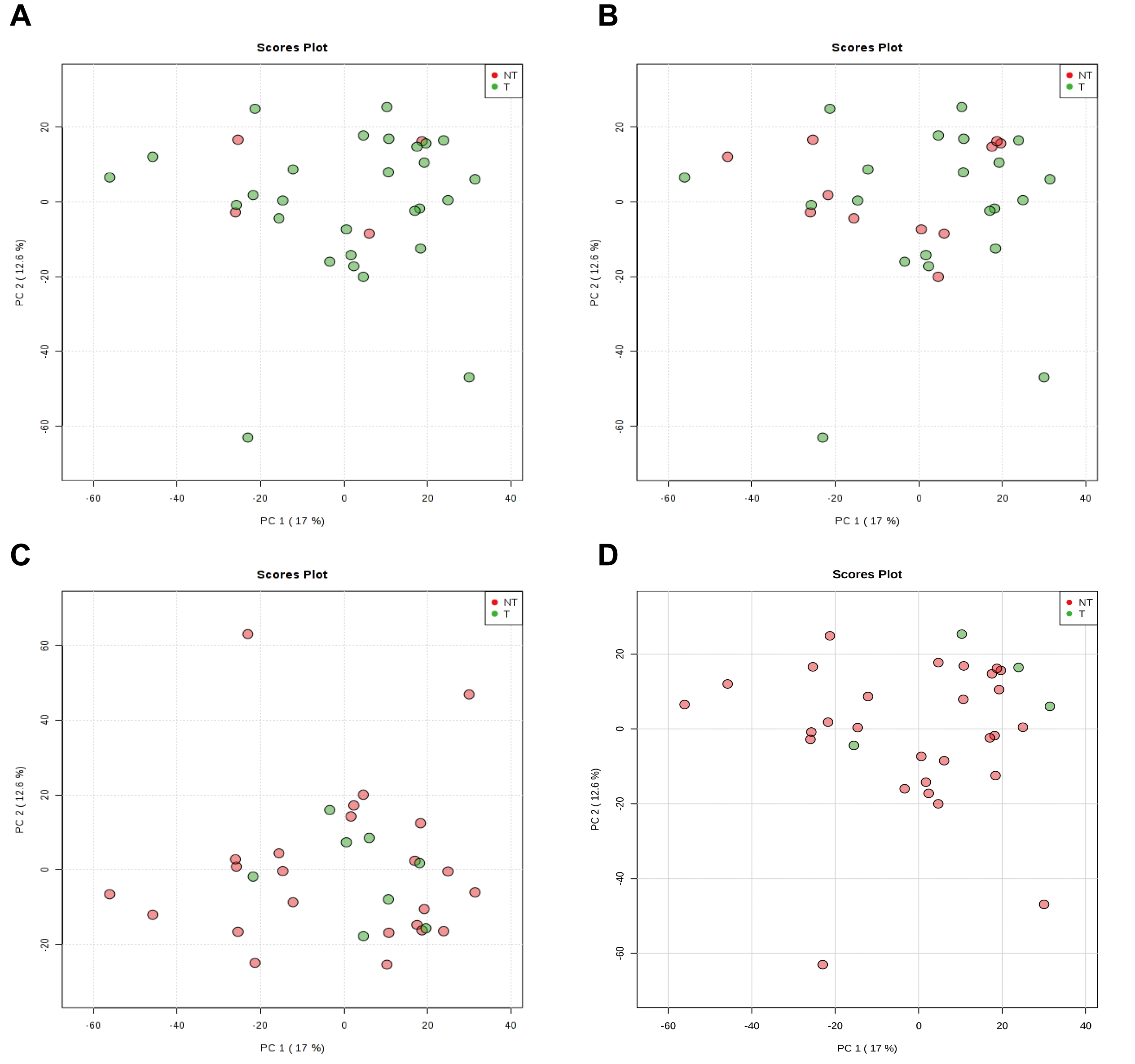


**Supplementary Figure S1** Relationship of fecal metabolism and medication treatments. Principal component analysis (PCA) of fecal metabolomics data from SLE patients with specific medicine (T) and patients with no specific medicine (NT) presented overall influences of glucocorticoid (A) hydroxycholorquine (B) cyclophosphamide (C) and leflunomide (D) on metabolomics of SLE patients.
